# Kaempferol and Kaempferide Attenuate Oleic Acid-Induced Lipid Accumulation and Oxidative Stress in HepG2 Cells

**DOI:** 10.3390/ijms22168847

**Published:** 2021-08-17

**Authors:** Fangfang Tie, Jin Ding, Na Hu, Qi Dong, Zhi Chen, Honglun Wang

**Affiliations:** 1 CAS Key Laboratory of Tibetan Medicine Research, Northwest Institute of Plateau Biology, Xining 810008, China; fftie@nwipb.cas.cn (F.T.); dingjin@163.com (J.D.); huna@nwipb.cas.cn (N.H.); qdong@nwipb.cas.cn (Q.D.); 2 Key Laboratory of Medicinal Animal and Plant Resources of Qinghai-Tibetan Plateau in Qinghai Province, Xining 810008, China; czi58@163.com

**Keywords:** NAFLD, kaempferol, kaempferide, lipid accumulation, oxidative stress, molecular docking

## Abstract

Nonalcoholic fatty liver disease (NAFLD) is one of the most common liver diseases which lacks ideal treatment options. Kaempferol and kaempferide, two natural flavonol compounds isolated from *Hippophae rhamnoides* L., were reported to exhibit a strong regulatory effect on lipid metabolism, for which the mechanism is largely unknown. In the present study, we investigated the effects of kaempferol and kaempferide on oleic acid (OA)-treated HepG2 cells, a widely used in vitro model of NAFLD. The results indicated an increased accumulation of lipid droplets and triacylglycerol (TG) by OA, which was attenuated by kaempferol and kaempferide (5, 10 and 20 μM). Western blot analysis demonstrated that kaempferol and kaempferide reduced expression of lipogenesis-related proteins, including sterol regulatory element-binding protein 1 (SREBP1), fatty acid synthase (FAS) and stearoyl-CoA desaturase 1 (SCD-1). Expression of peroxisome proliferator-activated receptor γ (PPARγ) and CCAAT enhancer binding proteins β (C/EBPβ), two adipogenic transcription factors, was also decreased by kaempferol and kaempferide treatment. In addition, western blot analysis also demonstrated that kaempferol and kaempferide reduced expression of heme oxygenase-1 (HO-1) and nuclear transcription factor-erythroid 2-related factor 2 (Nrf2). Molecular docking was performed to identify the direct molecular targets of kaempferol and kaempferide, and their binding to SCD-1, a critical regulator in lipid metabolism, was revealed. Taken together, our findings demonstrate that kaempferol and kaempferide could attenuate OA-induced lipid accumulation and oxidative stress in HepG2 cells, which might benefit the treatment of NAFLD.

## 1. Introduction

Nonalcoholic fatty liver disease (NAFLD) is one of the most common health problems worldwide. The main feature of NAFLD is lipid accumulation without significant alcohol consumption [1,2]. In clinic, NAFLD could be classified histologically into nonalcoholic fatty liver (NAFL) and nonalcoholic steatohepatitis (NASH) [3]. NAFLD is closely associated with type 2 diabetes, obesity, lipid metabolism dysfunction, atherosclerosis, hypertension and other metabolic disorders [3,4]. The pathogenesis of NAFLD remains unclear, and the “two-hit” hypothesis (recently updated as “multiple hits”) has been a leading theory [5]. The “first hit” is the excessive accumulation of triacylglycerol (TG) and free fatty acids [6], while the “second hit” refers to inflammation, oxidative stress and cellular apoptosis following the “first hit” [7].

There are three major resources of free fatty acids in the liver, diet, de novo lipogenesis and fatty acids released from adipose and other tissues [8]. De novo lipogenesis accounts for 30% of the liver fatty acid pool during fasting [9]. Enhanced efflux of free fatty acids into hepatocytes causes lipotoxicity, lipid metabolism disorder and excessive lipid accumulation, which promote pathogenesis of NAFLD [10]. Hepatic lipid metabolism could be regulated by lipogenesis-related proteins like SREBP1, FAS and SCD-1 [11,12]. Moreover, peroxisome proliferator-activated receptors (PPARs) play key roles in regulating lipid synthesis, storage, fatty-acid oxidation and adipogenesis [13]. C/EBPβ induces expression of PPARγ and C/EBPα, which form a positive feedback loop and contribute to the induction and maintenance of expression of adipocyte specific genes [14,15]. The accumulated fat is primarily stored as TG in the lipid droplets of adipose tissue [16,17]. Perilipin-1 belongs to the perilipin family and is a key coating protein on lipid droplets surface [18]. Targeting lipid metabolism associated proteins may facilitate the identification of promising drug candidates for prevent and/or treatment of NAFLD and other related metabolic disorders.

Kaempferol and kaempferide are two natural flavonols isolated from *Hippophae rhamnoides* L [19,20], a plant of the Elaeagnaceae family [21]. *Hippophae rhamnoides* L., is an edible medicinal plant used as a medicinal agent in traditional Chinese medicine and Tibetan medicine. *Hippophae rhamnoides* L., possesses biological properties of anti-tumor, anti-inflammation, anti-oxidation, hypoglycemia and hypotriglyceridemia [21,22,23,24], whereas kaempferol and kaempferide were suggested to be active in anti-cancer, anti-inflammation, anti-oxidation, anti-diabetes, anti-obesity and neuroprotection [25,26,27,28].

In the present study, we determined the effects of kaempferol and kaempferide on inhibiting oleic acid (OA)-induced lipid accumulation and oxidative stress in HepG2 cells.

## 2. Results

### 2.1. Induction of Steatosis in HepG2 Cells

**Figure 1:**
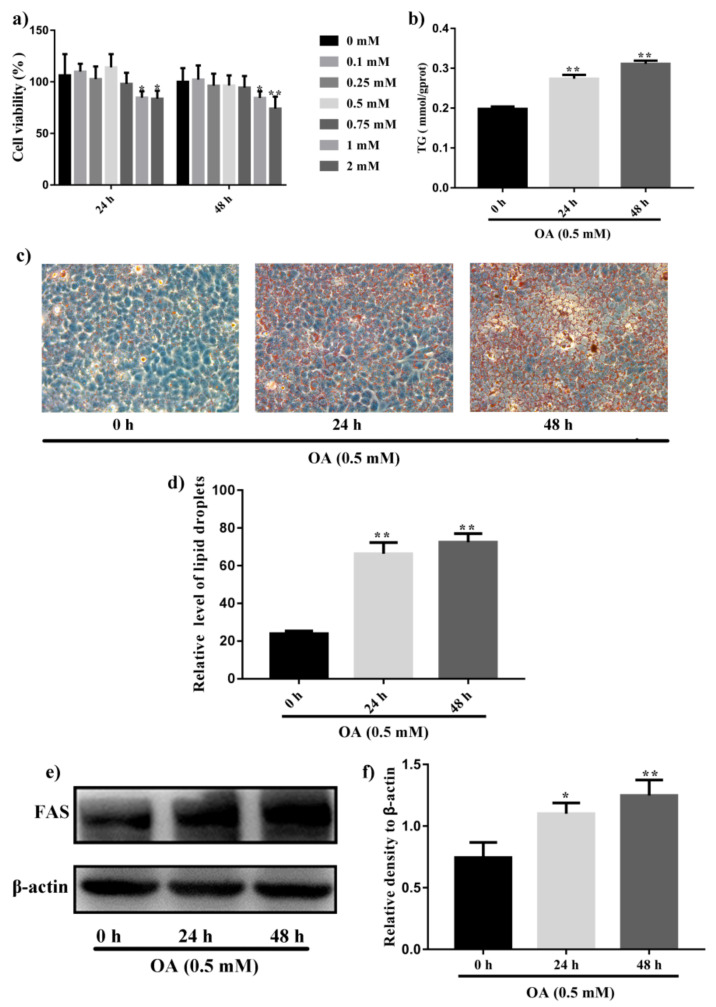
Induction of steatosis by OA in HepG2 cells. (**a**) SRB assay of cell viability of HepG2 cells treated with different concentration of OA for 24 h and 48 h. (**b**) Measurement of intracellular TG contents in HepG2 cells after incubation with 0.5 mM OA for 24 h and 48 h. (**c**) Oil red O staining to detect intracellular lipid droplets in HepG2 cells after treatment with 0.5 mM OA for 24 h and 48 h. (**d**) Quantitative analysis of intracellular lipid droplets accumulation in HepG2 cells. (**e**) Western blot analysis of expression of FAS in HepG2 cells after treatment with 0.5 mM OA for 24 h and 48 h. (**f**) Quantification results of the expression of FAS. Data were expressed as Mean ± SD of three independent experiments (*n* = 3). * *p* < 0.05 and ** *p* < 0.01, compared with HepG2 cells without OA treatment (0 h).

To model hepatic steatosis in vitro, HepG2 cells were treated with different concentrations of OA (0, 0.1, 0.25, 0.5, 0.75, 1 and 2 mM). As shown in Figure 1a, OA of less than 1 mM did not reduce cell viability after 24 h and 48 h incubation. However, reduction in HepG2 cells viability was observed when OA concentration was increased to more than 1 mM (*p* < 0.05). Therefore, OA of 0.5 mM was used to induce lipogenesis in HepG2 cells in the following studies. Lipid accumulation was investigated by oil red O staining. As shown in Figure 1c,d, large number of lipid droplets was formed in HepG2 cells after OA exposure for 48 h (*p* < 0.01), compared with untreated cells. Consistent with the results of oil red O staining, TG content in HepG2 cells was increased after OA incubation (Figure 1b). Furthermore, western blot analysis suggested increased expression of FAS (*p* < 0.05), a lipogenic protein, in HepG2 cells by OA treatment (Figure 1e,f). In summary, 0.5 mM OA could induce lipid accumulation in HepG2 cells without affecting cell viability.

**Figure 2:**
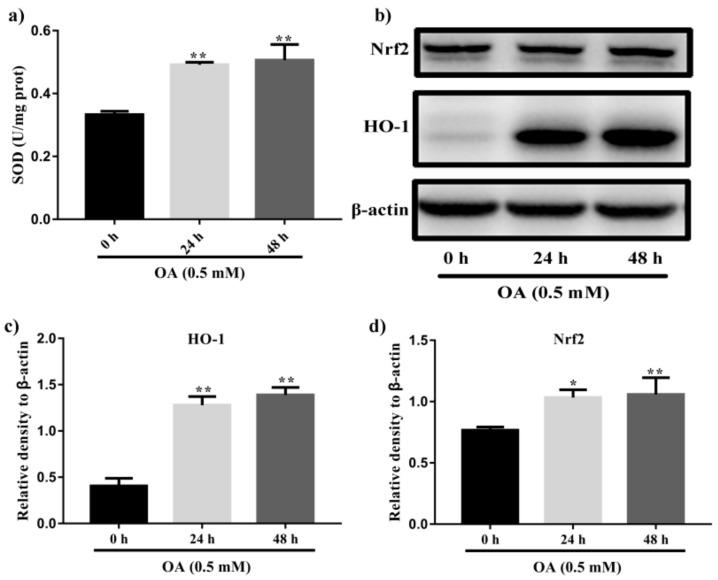
Induction of steatosis by OA in HepG2 cells. (**a**) Measurement of levels of SOD in HepG2 cells after incubation with 0.5 mM OA for 24 h and 48 h. (**b**) Western blot analysis of expression of Nrf2 and HO-1 in HepG2 cells after treatment with 0.5 mM OA for 24 h and 48 h. (**c**) Quantification results of the expression of HO-1. (**d**) Quantification results of the expression of Nrf2. Data were expressed as Mean ± SD of three independent experiments (*n* = 3). * *p* < 0.05 and ** *p* < 0.01, compared with HepG2 cells without OA treatment (0 h).

Recent studies suggested that the excess of oxidative stress could contribute to cellular injury and the pathogenesis of NAFLD. Hence, modulating antioxidant enzymes and oxidative stress may be significant for NAFLD treatment. SOD is important peroxidation indexes in NAFLD. As shown in Figure 2a, OA treatment for 48 h significantly increased the SOD content (*p* < 0.01). Concomitantly, HepG2 cells treated with 0.5 mM OA for 48 h prominently increase the protein levels of Nrf2 and HO-1 (*p* < 0.01, Figure 2b–d).

### 2.2. Effects of Kaempferol and Kaempferide on Cell Viability

**Figure 3:**
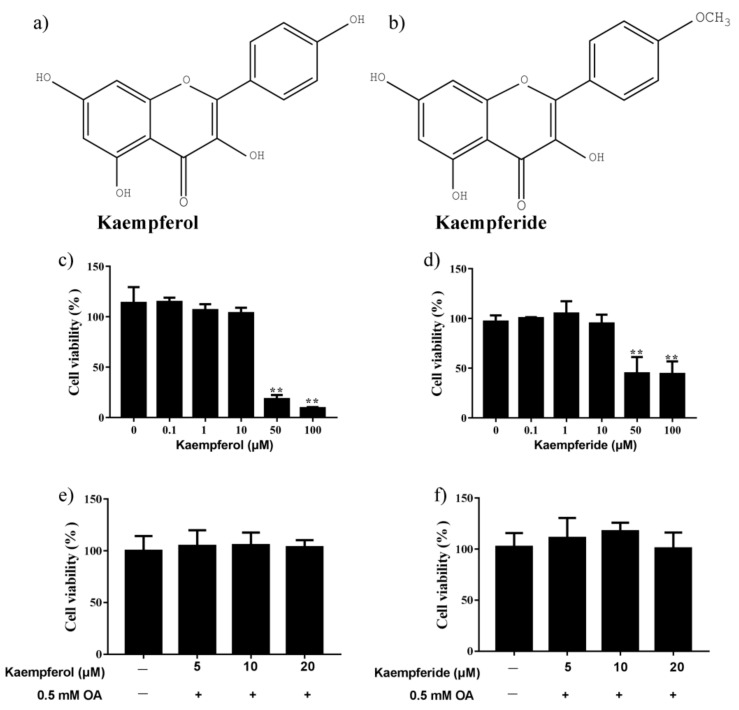
Changes in viability of HepG2 cells after incubation with kaempferol and kaempferide. (**a**) Chemical structure of kaempferol. (**b**) Chemical structure of kaempferide. (**c**) HepG2 cell viability after incubation with kaempferol. (**d**) HepG2 cell viability after incubation with kaempferide. (**e**) No change in HepG2 cell viability by co-incubation of OA and kaempferol for 48 h. (**f**) No change in HepG2 cell viability by co-incubation of OA and kaempferide for 48 h. Data were expressed as Mean ± SD of three independent experiments (*n* = 3). ** *p* < 0.01, compared with vehicle-treated control.

The structure of kaempferol and kaempferide were presented in Figure 3a,b. As shown in Figure 3c,d, kaempferol and kaempferide less than 10 μM did not change the viability of HepG2 cells. In contrast, kaempferol and kaempferide at 50 and 100 μM reduced HepG2 cell viability (*p* < 0.01) after incubation for 48 h. In addition, co-incubation of 0.5 mM OA with kaempferol and kaempferide (5, 10 and 20 μM) did not cause reduction in HepG2 cell viability, compared with vehicle-treated cells (Figure 3e,f), suggesting kaempferol and kaempferide do not affect cell viability of OA-treated HepG2 cells.

### 2.3. Kaempferol and Kaempferide Suppressed Lipid Accumulation in OA-Treated HepG2 Cells

**Figure 4:**
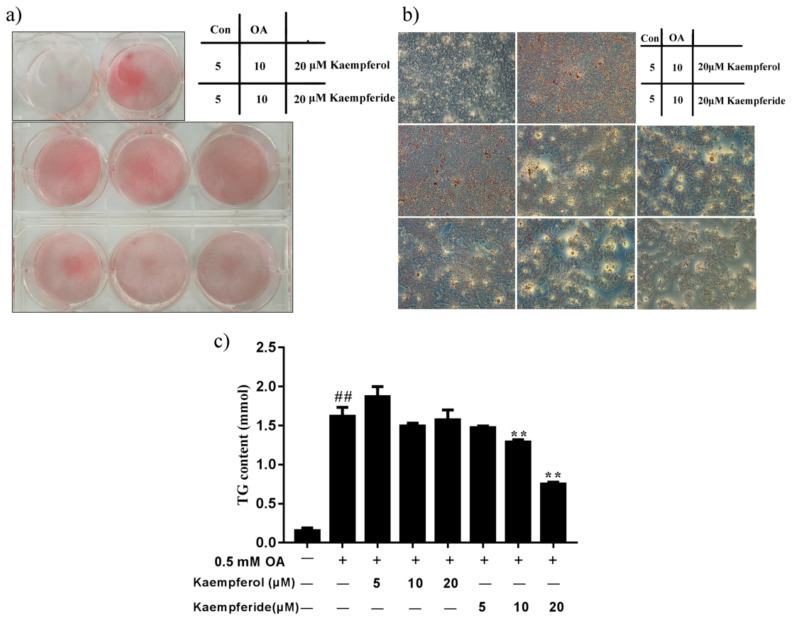
Kaempferol and kaempferide suppressed lipid accumulation in OA-induced HepG2 cells. HepG2 cells were incubated with different concentrations of kaempferol or kaempferide in the presence of 0.5 mM OA for 48 h. (**a**) Oil red O staining in the cultured HepG2 cells. (**b**) Visualization of intracellular lipid droplets in HepG2 cells under microscope (100× magnification). (**c**) Quantification of intracellular TG contents in HepG2 cells. Data were expressed as mean ± SD of three independent experiments (*n* = 3). ## *p* < 0.01, compared with vehicle-treated control cells (Con); ** *p* < 0.01, compared with OA-treated cells (OA).

To investigate whether kaempferol and kaempferide influence intracellular lipid accumulation, oil red O staining was performed. 0.5 mM OA caused prominent increase lipid droplets accumulation in HepG2 cells, compared with the control group (Figure 4a,b). Noticeably, incubation with kaempferol and kaempferide for 48 h reduced the accumulation of intracellular lipid droplets in a dose-dependent manner, compared with OA group. Moreover, kaempferide reduced the intracellular TG levels at concentration of 10 and 20 μM (*p* < 0.01), compared with the OA group (Figure 4c). Kaempferol treatment induced a trend of reduction in TG content, but statistical significance was not achieved. The results suggest that kaempferol and kaempferide attenuate OA-induced lipid accumulation in HepG2 cells.

### 2.4. Kaempferol and Kaempferide Decreased Expression of SREBP1, FAS and SCD-1 in OA-Treated HepG2 Cells

**Figure 5:**
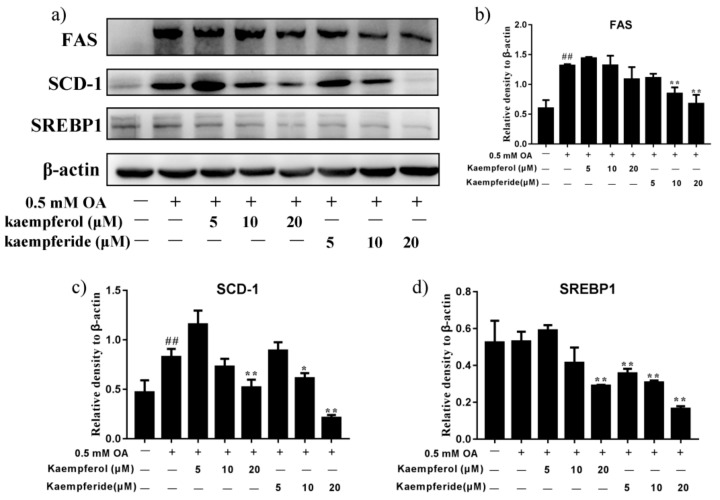
Kaempferol and kaempferide reduced expression of SREBP1, FAS and SCD-1 in OA-treated HepG2 cells. HepG2 cells were treated with different concentrations of kaempferol or kaempferide in the presence of 0.5 mM OA for 48 h followed by western blot analysis of expression of SREBP1, FAS and SCD-1. (**a**) Representative blots. (**b**) Quantification results of the expression of FAS. (**c**) Quantification results of the expression of SCD-1. (**d**) Quantification results of the expression of SREBP1. Data were expressed as mean ± SD of three independent experiments (*n* = 3). ## *p* < 0.01, compared with vehicle-treated control cells (Con); * *p* < 0.05 and ** *p* < 0.01, compared with OA-treated cells (OA).

To determine the underlying mechanism for the inhibitory effect of kaempferol and kaempferide on lipid accumulation, expression of lipogenesis-related proteins, SREBP1, FAS and SCD-1 were analyzed by western blot. As shown in Figure 5, kaempferide dose-dependently reduced the expression of SREBP1 in HepG2 cells (*p* < 0.01), compared with OA group. Reduction was also observed in expression of FAS and SCD-1 (*p* < 0.01), which was regulated by SREBP1. In contrast, treatment with kaempferol showed little effect on expression of SREBP1, FAS and SCD-1 (Figure 5). These findings suggest kaempferide may reduce lipid accumulation in OA-treated HepG2 cells via decreasing the expression of lipogenic proteins.

### 2.5. Kaempferol and Kaempferide Reduced Expression of PPARγ and C/EBPβ in OA-Treated HepG2 Cells

**Figure 6:**
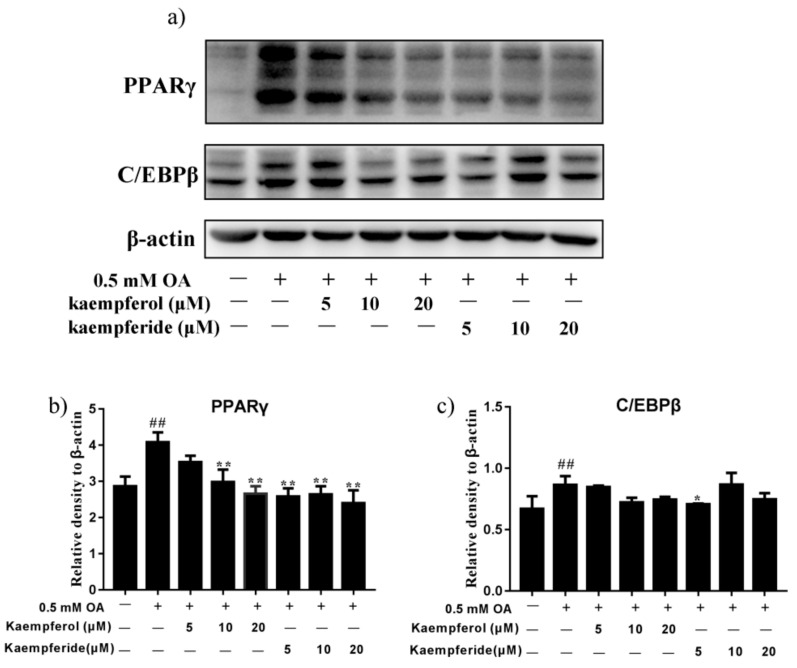
Kaempferol and kaempferide inhibited expression of PPARγ and C/EBPβ in OA-treated HepG2 cells. HepG2 cells were incubated with different concentrations of kaempferol or kaempferide in the presence of 0.5 mM OA for 48 h and followed by western blot analysis of the expression of PPARγ and C/EBPβ. (**a**) Representative blots. (**b**) Quantification results of the expression of PPARγ. (**c**) Quantification results of the expression of C/EBPβ. Data were expressed as mean ± SD of three independent experiments (*n* = 3). ## *p* < 0.01, compared with vehicle-treated control cells (Con); * *p* < 0.05 and ** *p* < 0.01, compared with OA-treated cells (OA).

To determine whether kaempferol and kaempferide regulate adipogenesis, protein levels of PPARγ and C/EBPβ, two adipogenic transcription factors, were determined by western blot. As shown in Figure 6, incubation with kaempferol (10 and 20 μM) and kaempferide (5, 10 and 20 μM), reduced the expression of PPARγ in HepG2 cells (*p* < 0.01), compared with OA group. A dose-dependent manner was observed for kaempferol. Moreover, incubation with kaempferol and kaempferide caused a trend of reduction in expression of C/EBPβ, although statistical significance was not realized. These results suggest kaempferol and kaempferide may reduce lipid accumulation by decreasing the expression of adipogenesis-related proteins.

### 2.6. Kaempferol and Kaempferide Reduced Expression of Perilipin-1 and Caveolin-1 in OA-Treated HepG2 Cells

**Figure 7:**
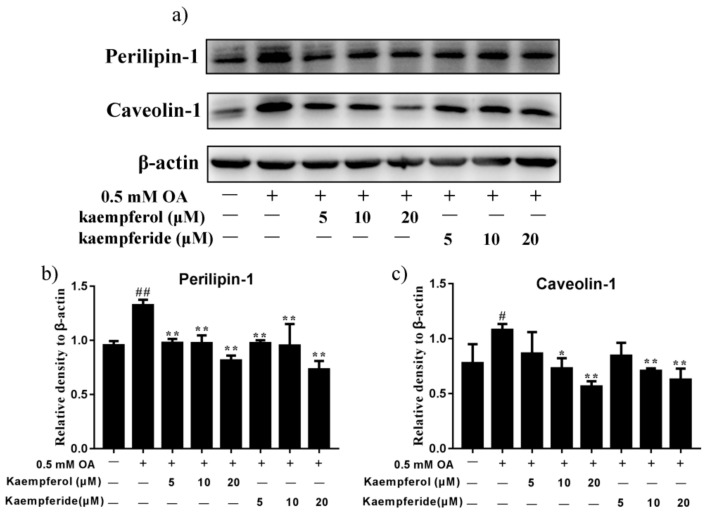
Kaempferol and kaempferide inhibited expression of Perilipin-1 and Caveolin-1 in OA-induced HepG2 cells. HepG2 cells were incubated with different concentrations of kaempferol or kaempferide in the presence of 0.5 mM OA for 48 h followed by western blot analysis of the expression of Perilipin-1 and Caveolin-1. (**a**) Representative blots. (**b**) Quantification results of the expression of Perilipin-1. (**c**) Quantification results of the expression of Caveolin-1. Data were expressed as mean ± SD of three independent experiments (*n* = 3). # *p* < 0.05 and ## *p* < 0.01, compared with vehicle-treated control cells (Con); * *p* < 0.05 and ** *p* < 0.01, compared with OA-treated cells (OA).

Expression of lipid droplet-associated proteins, Perilipin-1 and Caveolin-1, was determined by western blot. As shown in Figure 7, compared with OA group, incubation with kaempferol (5, 10 and 20 μM) and kaempferide (5, 10 and 20 μM) decreased the expression of Perilipin-1 in HepG2 cells (*p* < 0.01), with a dose-dependent effect being observed. Furthermore, kaempferol (10 and 20 μM) and kaempferide (10 and 20 μM) decreased the expression of Caveolin-1 (*p* < 0.05, Figure 7). These results suggest kaempferol and kaempferide may reduce lipid accumulation by decreasing the expression of lipid droplet proteins.

### 2.7. Kaempferol and Kaempferide Reduced Expression of Nrf2 and HO-1 in OA-Treated HepG2 Cells

**Figure 8:**
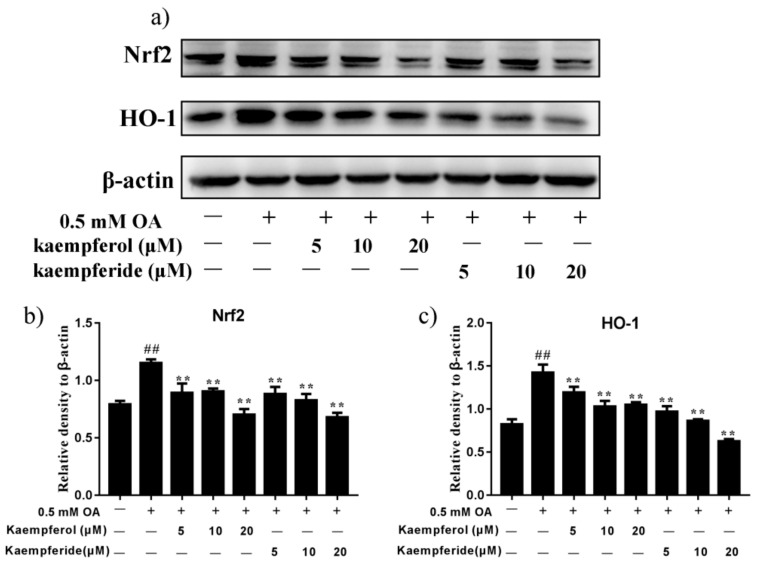
Kaempferol and kaempferide inhibited expression of Nrf2 and HO-1 in OA-induced HepG2 cells. HepG2 cells were incubated with different concentrations of kaempferol or kaempferide in the presence of 0.5 mM OA for 48 h followed by western blot analysis of the expression of Nrf2 and HO-1. (**a**) Representative blots. (**b**) Quantification results of the expression of Nrf2. (**c**) Quantification results of the expression of HO-1. Data were expressed as mean ± SD of three independent experiments. ## *p* < 0.01, compared with vehicle-treated control cells (Con); ** *p* < 0.01, compared with OA-treated cells (OA).

Under oxidative stress conditions, cells respond by activation of its antioxidant defense machinery to combat the cellular damage. We investigated the effect of kaempferol and kaempferide on oxidative stress by analyzing the changes in the Nrf2/HO-1 pathway. As shown in Figure 8, compared with OA group, incubation of kaempferol and kaempferide decreased the expression of Nrf2 and HO-1 in HepG2 cells (*p* < 0.01), with a dose-dependent effect being observed for both kaempferol and kaempferide. These results suggest kaempferol and kaempferide may reduce oxidative stress by decreasing the levels of Nrf2 and HO-1 expression.

### 2.8. Kaempferol and Kaempferide Bind to SCD-1

**Figure 9:**
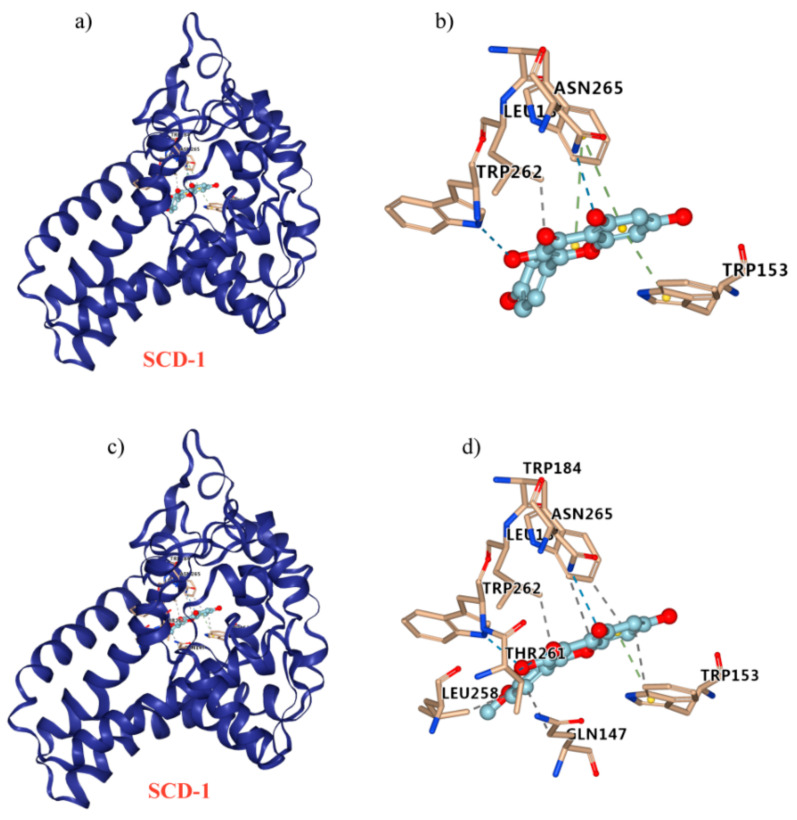
Molecular docking to predict the binding of kaempferol and kaempferide to SCD-1. (**a**) General overview of kaempferol docking into SCD-1. (**b**) Demonstration of the predicted binding conformation and corresponding interaction amino acid residues in (**a**). (**c**) General overview of kaempferide docking into SCD-1. (**d**) Demonstration of the predicted binding conformation and corresponding interaction amino acid residues in (**c**).

Molecular docking was performed to elucidate the possible interaction between kaempferol/kaempferide and SCD-1. Kaempferol was found to formed π-π stacking at residues Trp 184 and Trp153, hydrogen bond at residues Asn 265 and Trp 262, and hydrophobic interaction at residues Leu 185 (Figure 9a,b). For the interaction between kaempferide and SCD-1, π-π stacking was suggested at residues Trp153, hydrogen bond was suggested at residues Asn 265, Trp 262, and Thr 261, whereas hydrophobic interaction was suggested at residues Leu 185, Trp184, Leu 258, Trp 153, and Gln 147 (Figure 9c,d).

## 3. Discussion

NAFLD is a prevalent chronic liver disease characterized by lipid accumulation, hepatic steatosis, oxidative stress and hepatic apoptosis. Accordingly, inhibiting lipid accumulation and reducing oxidative stress should benefit the prevention and treatment of NAFLD. OA and palmitic acid (PA) are unsaturated fatty acid used to induce NAFLD model in HepG2 cells or L02 cell lines [29]. It is reported that OA exposure in cultured hepatocytes evoke hepatic steatosis, leading to insulin resistance, inflammation and even apoptosis [30]. Moreover, OA-treated HepG2 cells were used for determining the effect of interest compounds in lipid metabolism and NAFLD and the underlying mechanism [31]. In our study, lipid accumulation and the expression of FAS in HepG2 cells were increased by incubation with 0.5 mM OA for 48 h (Figure 1). In addition, increases in the level of SOD and the expression of Nrf2 and HO-1 also suggest the successful establishment of the NAFLD model (Figure 2).

Kaempferol and kaempferide, two natural flavonols, were abundantly present in *Hippophae rhamnoides* L., which have been used in food and traditional Chinese medicine for a long time. Both kaempferol and kaempferide have the parent nucleus of flavonoids, 2-phenyl-chromone. The major difference between the two is the substituents at the 4′ position of the B ring, which is phenolic hydroxyl group in kaempferol and methoxyl group in kaempferide. Recent studies found that kaempferol decreased lipid accumulation and attenuates PA-induced cellular lipotoxicity in β-cells [32]. Kaempferide decreased adipogenesis and the expression of adipogenic proteins in 3T3-L1 cells [33]. In the present study, we explored the molecular mechanisms for the lipid metabolism regulating effect of kaempferol and kaempferide in OA-induced HepG2 cells. Kaempferol and kaempferide did not inhibit the cell viability in HepG2 cells at tested concentrations (5, 10 and 20 μM) (Figure 3). Noticeably, kaempferol and kaempferide inhibited lipid accumulation in OA-treated HepG2 cells (Figure 4a,b). Retention of high level of lipids within hepatocytes, mostly in the form of TG, is required for the development of NAFLD [34]. Increased hepatic TG contents led to hepatocyte steatosis and induced lipotoxicity in the liver [35]. Our results showed that OA exposure increased TG content in HepG2 cells, while treatment with kaempferol and kaempferide attenuated this effect (Figure 4c).

AMPK is a conserved fuel-sensing enzyme that maintains hepatic energy balance via accelerating fatty acid β-oxidation and inhibiting adipogenesis/lipogenesis. AMPK directly inhibits the activation of SREBP1, a transcription factor that controls lipid metabolism [36]. The latter activates expression of FAS and SCD-1, two crucial proteins in hepatic fatty acid synthesis, to promote de novo lipogenesis [29]. SCD-1 is also closely associated with adipocyte cell differentiation and maturation and TG synthesis [31,37]. Our results demonstrated that kaempferol and kaempferide treatment in HepG2 cells ameliorated the OA-induced increase of SREBP1, FAS and SCD-1, with a dose-dependent manner being observed for kaempferide (Figure 5). These findings suggest kaempferol and kaempferide inhibit intracellular lipid accumulation by suppressing expression of lipogenic proteins. AMPK also inhibits adipogenesis-associated proteins, including C/EBPβ and PPARγ [38]. PPARγ is an adipogenic transcription factor that plays critical role in lipid synthesis, lipid storage and adipogenesis [39]. C/EBPβ is a transcription factor which by inducing expression of PPARγ and C/EBPα, promotes expression of adipocyte-specific genes. Our western blot analysis demonstrated that OA treatment enhanced the expression of PPARγ and C/EBPβ in HepG2 cells (Figure 6). Remarkably, this effect was attenuated by kaempferol and kaempferide. Kaempferide dose-dependently (5, 10 and 20 μM) decreased expression of PPARγ (Figure 6). In short, these results suggest the inhibition of expression of adipogenic proteins may contribute to the attenuating effect of kaempferol and kaempferide on hepatic lipid accumulation.

Nrf2 is an emerging regulator in cellular resistance to reactive oxidants and serves as a critical transcription factor in the regulation of expression of various cytoprotective genes, such as SOD and NQO1 [40,41]. Furthermore, activation of the transcription factor Nrf2 and HO-1 played vital roles in protecting cells from oxidative stress [42]. We hence investigated the antioxidants effects of kaempferol and kaempferide on OA-induced HepG2 cells. Our western blot analysis showed increased expression of Nrf2 and HO-1 by OA exposure, which effect was attenuated after treatment with kaempferol (5, 10 and 20 μM) and kaempferide (5, 10 and 20 μM). These results suggest kaempferol and kaempferide could attenuate oxidative stress in OA-treated HepG2 cells.

To provide a direct evidence for the role of SCD-1 in the inhibitory effect of kaempferol and kaempferide in lipid metabolism, we used molecular docking to predict the binding of kaempferol and kaempferide to SCD-1 [43,44]. Interestingly, we found that kaempferol and kaempferide could bind to SCD-1 (Figure 9). Compared with kaempferol, kaempferide may bind to SCD-1 in a more efficient way, in agreement with its stronger effects in reducing lipid accumulation and TG in OA-induced HepG2 cells (Figure 4).

Lipid droplets are the universal cell organelles for storage of neutral lipids. Lipid droplets consist of a triacylglycerol and sterol ester neutral lipid core, which is surrounded by a phospholipid monolayer containing a large number of proteins [45]. Perilipin-1 is a lipid droplet protein found in adipocytes and steroidogenic cells. Unphosphorylated perilipin-1 locates to the surface of intracellular lipid droplets to form a barrier and suppress lipolysis, while its phosphorylation initiates lipolysis [46]. Caveolin-1, perilipin-1 and the catalytic subunits of protein kinase A could form complex at the surface of lipid droplets to accelerate lipolysis [47]. Our western blot analysis showed that OA exposure increased the expression of Perilipin-1 and Caveolin-1 in HepG2 cells, while treatment with kaempferol and kaempferide attenuated the increase, in a dose-dependent manner (Figure 7). Compared to kaempferol, stronger inhibition effect was observed after treatment with kaempferide. These findings suggest kaempferol and kaempferide inhibit intracellular lipid accumulation by directly acting on the structural proteins of lipid droplets.

Many studies suggest, although not directly indicate, the incorporation of lipids into the cells. In the in vitro models of steatosis, the primary hepatic cells were treated with monounsaturated and saturated fatty acids [48], which seem to reproduce the key features of NAFLD in humans. Multiple free fatty acids were found to exert inherent toxic effects [49,50,51]. Among these, the saturated palmitic acid (PA, C16:0) and monounsaturated OA (C18:1) are the most abundant in hepatic triglycerides in both normal subjects and patients with NAFLD [52]. Literature data confirmed the induction of NAFLD in mice and in human hepatocytes exposed to PA and/or OA in primary cultures as well as in immortalized hepatocyte cell lines [53,54,55]. The incorporation of lipids (OA) into the HepG2 cells, treatment with kaempferol and kaempferide reduced TG content and decreased expression of PPARγ (Figure 4 and Figure 5). PA and OA have similar function in inducing NAFLD model in vitro. Thus, we think when incorporation of lipids (PA) into the HepG2 cells, treatment with kaempferol and kaempferide also reduced TG content and decreased expression of lipogenic proteins.

## 4. Materials and Methods

### 4.1. Chemicals and Reagents

Kaempferol and kaempferide were isolated from *Hippophae rhamnoides* L., as previously described [20,56]. OA, oil red O and sulforhodamine B (SRB) were purchased from Sigma-Aldrich (St. Louis, MO, USA). Dulbecco’s Modified Eagle Medium (DMEM) was purchased from Gibco (Carlsbad, CA, USA). Fetal Bovine Serum (FBS) was from Zhejiang Tianhang Biological Technology Co., Ltd. Kits of measurement of triglyceride (TG) and superoxide dismutase (SOD) were obtained from Nanjing Jiancheng Bioengineering Institute (Nanjing, China). BCA assay kit and protein lysate buffer were obtained from Beyotime (Shanghai, China). Polyclonal antibodies against FAS, SCD-1, SREBP1, PPARγ, C/EBPα, Perilipin-1, Caveolin-1, Nrf2, HO-1 and β-actin were obtained from Cell Signaling Technology (Danvers, MA, USA).

### 4.2. Culture of HepG2 Cells

HepG2 is a human liver cancer cell line, which was purchased from Cell Resource Center of Shanghai Institute of Life Sciences, Chinese Academy of Science (Shanghai, China). Cells were cultured in DMEM supplemented with 10% FBS in atmosphere of 5% CO_2_ at 37 °C.

### 4.3. Cell Viability Assay

The SRB method was used to assess cell viability. Firstly, HepG2 cells were cultured in a 96-well plate at density of 1 × 10^5^ cells per mL. To determine the toxicity, HepG2 cells were exposed to different concentrations of OA (0–2 mM) for 24 and 48 h, or kaempferol (0–100 μM) or kaempferide (0–100 μM) for 48 h. To determine the lipid accumulation inhibiting effect of kaempferol and kaempferide, HepG2 cells were incubated with kaempferol (5, 10 and 20 μM) or kaempferide (5, 10 and 20 μM) in the presence of 0.5 mM OA for 48 h. After incubation, the medium was then replaced with 25 μL of 50% cold trichloroacetic acid and incubated at 4 °C for 1 h. The plates were washed with distilled water for five times, and air-dried at room temperature for 1 h. After that, the cells were stained with 70 μL of 0.4% SRB for 30 min in the dark. Dyed cells were washed four times with 1% acetic acid and the protein-bound dye was dissolved by adding 100 μL of 10 mM Tris base buffer and shaking on an orbital shaker for 20 min. Finally, absorbance at 540 nm was measured with microplate reader (Molecular Devices, CA, USA).

### 4.4. Oil Red O Staining

Oil Red O staining was used to measure lipid droplet content in HepG2 cells. The HepG2 cells were cultured in 6-well plates at a density of 5 × 10^4^ cells per mL, with 0.5 mM OA being added in the medium. Cells were treated with or without different concentrations (5, 10 and 20 μM) of kaempferol or kaempferide for 48 h. After treatment, cells were washed twice with PBS before fixation with 4% paraformaldehyde for 30 min in darkness. Subsequently, treated cells were stained with oil red O solution for 1 h. The lipid droplets were observed using an inverted light microscope (Olympus, Tokyo, Japan). Finally, the cells were incubated with 100% isopropanol solution to dissolve the lipid-bound red dye and optical density was measured at 520 nm.

### 4.5. Measurement of the TG Content

A commercial kit was used to determine intracellular TG content. The HepG2 cells were cultured in 6-well plates at a density of 5 × 10^4^ cells per mL, in the presence of 0.5 mM OA. The cells were incubated with or without the presence of different concentrations (5, 10 and 20 μM) of kaempferol or kaempferide for 48 h. The treated cells were dissociated by trypsinization. Dissociated cells were homogenized by ultrasonication for 5 min on ice, after which TG contents were measured using a commercial kit according to the manufacturer’s protocol.

### 4.6. Measurement of the Levels of SOD

A commercial kit was used to determine the levels of SOD. The HepG2 cells were cultured in 6-well plates at a density of 5 × 10^4^ cells per mL, in the presence of 0.5 mM OA. The cells were incubated with or without presence of different concentrations (5, 10 and 20 μM) of kaempferol or kaempferide for 48 h. The cell morphology was observed under an inverted microscope. Furthermore, the levels of SOD were measured using a commercial kit according to the manufacturer’s protocol.

### 4.7. Western Blot Analysis

Cells were washed with cold PBS and homogenized in RIPA buffer containing protease inhibitors. The homogenates were centrifuged at 12,000× *g* for 15 min at 4 °C and the supernatant was collected. Samples of 20 μg proteins were separated on 10% SDS polyacrylamide gel and transferred to PVDF membrane (Millipore. Billerica, MA, USA). The membranes were blocked with skim milk solution at room temperature for 1 h before incubation with primary antibodies overnight at 4 °C. The membrane was washed twice with TBST buffer followed by incubation with secondary antibody for 1 h. β-actin was used as internal control. Blots were visualized with 5200 Multi Luminescent imaging systems (Tanon, Shanghai, China). The intensity of bands was analyzed with Image J (NIH).

### 4.8. Molecular Docking Studies

The crystal structures of SCD-1 (PDB ID: 4zyo) was downloaded from RCSB Protein Data Bank and the 3D structures of kaempferol and kaempferide were downloaded from NCBI PubChem Compound. Processing of the proteins and compounds and molecular docking were performed in Autodock Vina.

### 4.9. Statistical Analysis

Date analysis was performed with GraphPad Prism 7.0 (GraphPad Software, Inc., San Diego, CA, USA). Results were presented as Mean ± Standard deviation (SD) from three independent experiments. Comparisons between groups were performed with unpaired Student’s t-test (two groups) or one-way ANOVA (three or more groups). *p* < 0.05 was considered to be statistically significant.

## 5. Conclusions

In summary, our findings demonstrate that kaempferol and kaempferide could attenuate OA-induced lipid accumulation and oxidative stress in HepG2 cells. The mechanism might be the inhibiting effect of kaempferol and kaempferide on the expression of lipogenic and adipogenic proteins, lipid droplet proteins, and oxidative stress-related proteins. Our molecular docking analysis suggests kaempferol and kaempferide could directly bind to SCD-1, a lipogenic protein. In short, we anticipate that kaempferol and kaempferide possess potential as drug candidates for the prevention and/or treatment of NAFLD.

## Data Availability

Date is contained within the article.

## References

[1] Zhang E., Song X., Yin S., Fan L., Ye M., Hu H. (2017). Glycycoumarin prevents hepatic steatosis through activation of adenosine 5,-monophosphate (AMP)-activated protein kinase signaling pathway and up-regulation of BTG1/Tob-1. J. Funct. Foods..

[2] Zhu X., Xiong T., Liu P., Guo X., Xiao L., Zhou F., Tang Y., Yao P. (2018). Quercetin ameliorates HFD-induced NAFLD by promoting hepatic VLDL assembly and lipophagy via the IRE1a/XBP1s pathway. Food Chem. Toxicol..

[3] Younossi Z., Anstee Q.M., Marietti M., Hardy T., Henry L., Eslam M., George J., Bugianesi E. (2018). Global burden of NAFLD and NASH: Trends, predictions, risk factors and prevention. Nat. Rev. Gastroenterol. Hepatol..

[4] Radaelli M., Martucci F., Perra S., Accornero S., Castoldi G., Lattuda G., Manzoni G., Perseghin G. (2018). NAFLD/NASH in patients with type 2 diabetes and related treatment options. J. Endocrinol. Invest..

[5] Day C.P., James O.F. (1998). Steatohepatitis: A tale of two “hits”?. Gastroenterology.

[6] Jin X.L., Wang K., Li Q.Q., Tian W.L., Xue X.F., Wu L.M., Hu F.L. (2017). Antioxidant and anti-inflammatory effects of Chinese propolis during palmitic acid-induced lipotoxicity in cultured hepatocytes. J. Funct. Foods.

[7] Zhang D.D., Zhang J.G., Wu X., Liu Y., Gu S.Y., Zhu G.H., Wang Y.Z., Liu G.L., Li X.Y. (2015). Nuciferine downregulates Per-Arnt-Sim kinase expression during its alleviation of lipogenesis and inflammation on oleic acid-induced hepatic steatosis in HepG2 cells. Front. Pharmacol..

[8] Cohen J.C., Horton J.D., Hobbs H.H. (2011). Human fatty liver disease: Old questions and new insights. Science.

[9] Gluchowski N.L., Becuwe M., Walther T.C., Farese R.V. (2017). Lipid droplets and liver disease: From basic biology to clinical implications. Nat. Rev. Gastroenterol. Hepatol..

[10] Guo L., Kang J.S., Park Y.H., Je B.I., Lee Y.J., Kang N.J., Park S.Y., Hwang D.Y., Choi Y.W. (2020). S-petasin inhibits lipid accumulation in oleic acid-induced HepG2 cells through activation of the AMPK signaling pathway. Food Funct..

[11] Lee H.J., Le B., Lee D.R., Choi B.K., Yang S.H. (2018). Extract (CQR-300) inhibits lipid accumulation by downregulating adipogenesis and lipogenesis in 3T3-L1 cells. Toxicol. Rep..

[12] Qi G., Guo R., Tian H., Li L., Liu H., Mi Y., Liu X. (2018). Nobiletin protects against insulin resistance and disorders of lipid metabolism by reprogramming of circadian clock in hepatocytes. Biochim. Biophys. Acta Mol. Cell Biol. Lipids.

[13] Farmer S.R. (2005). Regulation of PPARgamma activity during adipogenesis. Int. J. Obes..

[14] Ntambi J.M., Young-Cheul K. (2000). Adipocyte differentiation and gene expression. J. Nutr..

[15] Scott M.A., Nguyen V.T., Levi B., James A.W. (2011). Current methods of adipogenic differentiation of mesenchymal stem cells. Stem Cells Dev..

[16] Martin S., Parton R.G. (2006). Lipid droplets: A unified view of a dynamic organelle. Nat. Rev. Mol. Cell Biol..

[17] Schulze R.J., Sathyanarayan A., Mashek D.G. (2017). Breaking fat: The regulation and mechanisms of lipophagy. Biochim. Biophys. Acta Mol. Cell Biol. Lipids.

[18] Walther T.C., Farese R.V. (2012). Lipid droplets and cellular lipid metabolism. Annu. Rev. Biochem..

[19] Yue M.-E., Jiang T.-F., Shi Y.-P. (2004). Fast determination of flavonoids in Hippophae rhamnoides and its medicinal preparation by capillary zone electrophoresis using dimethyl-beta-cyclodextrin as modifier. Talanta.

[20] Zhang Q., Cui H. (2005). Simultaneous determination of quercetin, kaempferol, and isorhamnetin in phytopharmaceuticals of *Hippophae rhamnoides* L. by high-performance liquid chromatography with chemiluminescence detection. J. Sep. Sci..

[21] Pundir S., Garg P., Dviwedi A., Ali A., Kapoor V.K., Kapoor D., Kulshrestha S., Lal U.R., Negi P. (2021). Ethnomedicinal uses, phytochemistry and dermatological effects of *Hippophae rhamnoides* L.: A review. J. Ethnopharma..

[22] Koyama T., Taka A., Togashi H. (2009). Effects of a herbal medicine, Hippophae rhamnoides, on cardiovascular functions and coronary microvessels in the spontaneously hypertensive stroke-prone rat. Clin. Hemorheol. Microcirc..

[23] Saeidi K., Alirezalu A., Akbari Z. (2016). Evaluation of chemical constitute, fatty acids and antioxidant activity of the fruit and seed of sea buckthorn (*Hippophae rhamnoides* L.) grown wild in Iran. Nat. Prod. Res..

[24] Chauhan A.S., Negi P.S., Ramteke R.S. (2007). Antioxidant and antibacterial activities of aqueous extract of Seabuckthorn (*Hippophae rhamnoides*) seeds. Fitoterapia.

[25] Kashyap D., Sharma A., Tuli H.S., Sak K., Punia S., Mukherjee T.K. (2017). Kaempferol—A dietary anticancer molecule with multiple mechanisms of action: Recent trends and advancements. J. Funct. Foods..

[26] Zang Y., Zhang L., Igarashi K., Yu C. (2015). The anti-obesity and anti-diabetic effects of kaempferol glycosides from unripe soybean leaves in high-fat-diet mice. Food Funct..

[27] Lee Y.J., Choi H.S., Seo M.J., Jeon H.J., Kim K.J., Lee B.Y. (2015). Kaempferol suppresses lipid accumulation by inhibiting early adipogenesis in 3T3-L1 cells and zebrafish. Food Funct..

[28] Tang H., Zeng Q., Tang T., Wei Y., Pu P. (2021). Kaempferide improves glycolipid metabolism disorder by activating PPARγ in high-fat-diet-fed mice. Life Sci..

[29] Chang Y.H., Chen Y.L., Huang W.C., Liou C.J. (2018). Fucoxanthin attenuates fatty acid-induced lipid accumulation in FL83B hepatocytes through regulated Sirt1/AMPK signaling pathway. Biochem. Biophys. Res. Commun..

[30] He Y., Yang W., Gan L., Liu S., Ni Q., Bi Y., Han T., Liu Q., Chen H., Hu Y. (2021). Silencing HIF-1α aggravates non-alcoholic fatty liver disease in vitro through inhibiting PPAR-α/ANGPTL4 singling pathway. Gastroenterol. Hepatol..

[31] Zhao N., Yang S., Jia Y., Sun B., He B., Zhao R. (2018). Maternal betaine supplementation attenuates glucocorticoid-induced hepatic lipid accumulation through epigenetic modification in adult offspring rats. J. Nutr. Biochem..

[32] Varshney R., Varshney R., Mishra R., Gupta S., Sircar D., Roy P. (2018). Kaempferol alleviates palmitic acid-induced lipid stores, endoplasmic reticulum stress and pancreatic β-cell dysfunction through AMPK/mTOR-mediated lipophagy. J. Nutr. Biochem..

[33] Kumkarnjana S., Suttisri R., Nimmannit U., Sucontphunt A., Khongkow M., Koobkokkruad T., Vardhanabhuti N. (2019). Flavonoids kaempferide and 4,20-dihydroxy-40,50,60-trimethoxychalcone inhibit mitotic clonal expansion and induce apoptosis during the early phase of adipogenesis in 3T3-L1 cells. J. Integr. Med..

[34] Angulo P. (2002). Nonalcoholic fatty liver disease. N. Engl. J. Med..

[35] Kawano Y., Cohen D.E. (2013). Mechanisms of hepatic triglyceride accumulation in non-alcoholic fatty liver disease. J. Gastroenterol..

[36] Yuan H.X., Xiong Y., Guan K.L. (2013). Nutrient sensing, metabolism, and cell growth control. Mol. Cell..

[37] Jensen-Urstad A.P.L., Semenkovich C.F. (2012). Fatty acid synthase and liver triglyceride metabolism: Housekeeper or messenger?. Biochim. Biophys. Acta.

[38] Feng S., Reuss L., Wang Y. (2016). Potential of natural products in the inhibition of adipogenesis through regulation of PPARγ expression and/or its transcriptional activity. Molecules.

[39] Ament Z., Masoodi M., Griffin J.L. (2012). Applications of metabolomics for understanding the action of peroxisome proliferator-activated receptors (PPARs) in diabetes, obesity and cancer. Genome. Med..

[40] Cichoz-Lach H., Michalak A. (2014). Oxidative stress as a crucial factor in liver diseases. World J. Gastroenterol..

[41] Zhu H., Itoh K., Yamamoto M., Zweier J.L., Li Y. (2005). Role of Nrf2 signaling in regulation of antioxidants and phase 2 enzymes in cardiac fibroblasts: Protection against reactive oxygen and nitrogen species-induced cell injury. FEBS Lett..

[42] Chen J., Tian J., Ge H. (2017). Effects of tetramethylpyrazine from Chinese black vinegar on antioxidant and hypolipidemia activities in HepG2 cells. Food Chem. Toxicol..

[43] Da Silva-Junior E.F., Barcellos Franca P.H., Ribeiro F.F., Bezerra Mendonca-Junior F.J., Scotti L., Scotti M.T., de Aquino T.M., de Araujo-Junior J.X. (2018). Molecular docking studies applied to a dataset of cruzain inhibitors. Curr. Comput. Aided Drug Des..

[44] Roy N., Narayanankutty A., Nazeem P.A., Valsalan R., Babu T.D., Mathew D. (2016). Plant phenolics ferulic acid and p-coumaric acid inhibit colorectal cancer cell proliferation through EGFR down-regulation. Asian Pac. J. Cancer Prev..

[45] Walther T.C., Chung J., Farese R.V. (2017). Lipid droplet biogenesis. Annu. Rev. Cell. Dev. Biol..

[46] Jackson C.L. (2019). Lipid droplet biogenesis. Curr. Opin. Cell. Biol..

[47] Xu S., Zhang X., Liu P. (2018). Lipid droplet proteins and metabolic diseases. Biochim. Biophys. Acta Mol. Basis Dis..

[48] Feldstein A.E., Canbay A., Guicciardi M.E., Higuchi H., Bronk S.F., Gores G.J. (2003). Diet associated hepatic steatosis sensitizes to Fas mediated liver injury in mice. J. Hepatol..

[49] Malhi H., Bronk S.F., Werneburg N.W., Gores G.J. (2006). Free fatty acids induce JNK-dependent hepatocyte lipoapoptosis. J. Biol Chem..

[50] Donato M.T., Lahoz A., Jiménez N., Pérez G., Serralta A., Mir J., Castell J.V., Gómez-Lechón M.J. (2006). Potential impact of steatosis on cytochrome P450 enzymes of human hepatocytes isolated from fatty liver grafts. Drug Metab. Dispos..

[51] Wang D., Wei Y., Pagliassotti M.J. (2006). Saturated fatty acids promote endoplasmic reticulum stress and liver injury in rats with hepatic steatosis. Endocrinology.

[52] Araya J., Rodrigo R., Videla L.A., Thielemann L., Orellana M., Pettinelli P., Poniachik J. (2004). Increase in long-chain polyunsaturated fatty acid n—6/n—3 ratio in relation to hepatic steatosis in patients with non-alcoholic fatty liver disease. Clin. Sci..

[53] Tang Y., Bian Z., Zhao L., Liu Y., Liang S., Wang Q., Han X., Peng Y., Chen X., Shen L. (2011). Interleukin-17 exacerbates hepatic steatosis and inflammation in non-alcoholic fatty liver disease. Clin. Exp. Immunol..

[54] Gómez-Lechón M.J., Donato M.T., Martínez-Romero A., Jiménez N., Castell J.V., O’Connor J.E. (2007). A human hepatocellular in vitro model to investigate steatosis. Chem. Biol. Interact..

[55] Mantzaris M.D., Tsianos E.V., Galaris D. (2011). Interruption of triacylglycerol synthesis in the endoplasmic reticulum is the initiating event for saturated fatty acid-induced lipotoxicity in liver cells. FEBS J..

[56] Jia Q., Zhang S., Zhang H., Yang X., Cui X., Su Z., Hu P. (2020). A comparative study on polyphenolic composition of berries from the Tibetan plateau by UPLC-Q-orbitrap MS system. Chem. Biodivers..

